# Deployment and Validation of a Smart System for Screening of Language Disorders in Primary Care

**DOI:** 10.3390/s130607522

**Published:** 2013-06-10

**Authors:** María Luisa Martín-Ruiz, Miguel Ángel Valero Duboy, Iván Pau de la Cruz

**Affiliations:** Departamento de Ingeniería y Arquitecturas Telemáticas, Universidad Politécnica de Madrid, Carretera de Valencia km. 7, Madrid 28031, Spain; E-Mails: mavalero@diatel.upm.es (M.A.V.D.); ipau@diatel.upm.es (I.P.C.)

**Keywords:** intelligent healthcare, knowledge management, early attention, e-health, language disorders

## Abstract

Neuro-evolutive development from birth until the age of six years is a decisive factor in a child's quality of life. Early detection of development disorders in early childhood can facilitate necessary diagnosis and/or treatment. Primary-care pediatricians play a key role in its detection as they can undertake the preventive and therapeutic actions requested to promote a child's optimal development. However, the lack of time and little specific knowledge at primary-care avoid to applying continuous early-detection anomalies procedures. This research paper focuses on the deployment and evaluation of a smart system that enhances the screening of language disorders in primary care. Pediatricians get support to proceed with early referral of language disorders. The proposed model provides them with a decision-support tool for referral actions to trigger essential diagnostic and/or therapeutic actions for a comprehensive individual development. The research was conducted by starting from a sample of 60 cases of children with language disorders. Validation was carried out through two complementary steps: first, by including a team of seven experts from the fields of neonatology, pediatrics, neurology and language therapy, and, second, through the evaluation of 21 more previously diagnosed cases. The results obtained show that therapist positively accepted the system proposal in 18 cases (86%) and suggested system redesign for single referral to a speech therapist in three remaining cases.

## 1. Introduction

Pediatricians that work in the Spanish Healthcare System (SHS) are primary care physicians who provide care in health centers to children between the ages of 0 and 14. These professionals mainly use information systems that fulfill the WONCA standards [1] with different degrees of compliance. A widely deployed system is OMI, a software solution aligned with WONCA to register medical data that does not cover key aspects to follow-up the children's neuro-evolutive and social development.

The Early Intervention Program (New York State Department of Health [2]) reinforces the aspect of permanent monitoring of children: “The early years of a child's life are very important. In these years, children grow quickly and have so much to learn. Developmental delay means that a child has not attained developmental milestones expected for the child's age adjusted for prematurity in one or more of the following areas of development: cognitive, physical (including vision and hearing), communication, social-emotional, or adaptive development”. Furthermore, the Speech-language pathology is included as a key area for the Early Intervention Official [2].

As stated in the Spanish white paper on early attention [3], pediatricians must screen and correctly refer children with potential disorders towards diagnostic and monitoring centers. Present rates of detection of development disorders are lower than their real incidence [4], which means that early identification of children with such disorders remains a pending task. In certain cases, language disorders are the first symptoms a child will manifest of a possible development disorder [5] and expressive language delay is present in many cases of preterm infants [6].

However, in clinical practice, pediatricians usually lack enough time to perform proper screening of the children's neuro-evolutive development, and their background in disabilities is not always as complete as it would be desirable [7]. For these reasons, a scientific and technological contribution to this problem is the deployment and validation a Knowledge Based System (KBS) in primary care, capable of providing pediatricians with valuable support to enable efficient screening of children's disorders. A smart screening system in such healthcare scenario does require the construction of a substantial and comprehensive Knowledge Base (KB) to test effective approaches to this problem. This research paper describes the process of building such a KBS based on multi-disciplinary work with experts and the analysis of 21 actual proven cases diagnosed in the Language Intervention Center (LIC). Moreover, the result stage presents the KBS verification with 21 cases previously diagnosed in the LIC.

Section 2 provides details on the background of this research from two complementary perspectives: the standpoint of experts in the detection of neurological disorders in childhood, and application of expert systems in medicine. The next section explains the methodology used in the development of the KBS for primary pediatric care. A description is provided of the process of system knowledge education with Knowledge Based System tools, the procedures for extracting information managed by experts and the stages of construction of the ontology used in the reasoning model. Sections 4 and 5 present the development and the deployment of the system, respectively. Section 6 reports the main results of the research which includes two complementary approaches for the platform validation: firstly, through the participation of seven experts from the fields of neonatology, pediatrics, neurology and language therapy, and, second, by evaluating 21 previously diagnosed cases. Section 7 concludes the paper and present future work directions.

## 2. Background

### 2.1. Early Detection Systems of Neurological Disorders in Children

The Spanish white paper on early attention emphasizes the importance of intervention for the transitory nature of a certain development disorder [3]. Such disorders must be considered a significant deviation from the “course” of development resulting from health or relational events that compromise biological, psychological and social development. Hence, detection of possible alterations in child development is essential for early attention, as it will foster the activation of a number of requested mechanisms of action.

The utilization of smart systems in primary care can improve the detection of neurological disorders in children by enabling the prevention of added pathologies and fostering functional improvements that allow a more adaptive relationship between a child and his or her surroundings. Narbona highlights that a delay in the correct acquisition of speech and language is a first alarm sign of a future neurological disorder although it is not the only symptom to have into account to diagnose a neurological disorder [5]. The paper published by Nelson *et al.* explains why the correct acquisition of language is of vital importance [8], while Fejerman stresses that a complete neurological and pediatric evaluation can reveal related developmental disorders, starting with detection of a language disorder [9]. Although medical procedures are available to detect a number of neurological disorders in children [4,10–12] these procedures are difficult to apply in primary pediatric care, as many require significant time and specialized knowledge. The review in this research found no solutions that exploit the potential of Information Systems in combination with artificial intelligence to provide pediatricians with efficient and effective assistance in the early detection of these disorders.

The systematic review of systems that may improve early attention on children was elaborated in this research according to the followings related sources: PubMed, JAMIA and ModernMedicine. The most valuable keywords used were “early attention”, “language disorders” and “eHealth”.

- Przybylski *et al.* presented a study tested the influence of external rhythmic auditory stimulation (*i.e.*, musical rhythm) on syntax processing in children with specific language impairment (SLI) and dyslexia. Results points to potential avenues in using rhythmic structures (even in nonverbal materials) to boost linguistic structure processing [13].
- Naddy described a pediatric early warning tool used with routine nursing observations will alert staff to the need for increased monitoring, the support of an associated outreach team or emergency medical attention [14].
- McLellan and Connor describe the modification of a pediatric early warning scoring system for cardiovascular patients, the implementation of the tool, and its companion Escalation of Care Algorithm on an inpatient pediatric cardiovascular unit [15].
- Parshuram, Hutchison and Middaugh developed and validated a simple bedside score to quantify severity of illness in hospitalized children [16].
- Haines, Perrott and Weir developed and evaluated a physiologically based system for the identification of acutely ill children in hospital environments [17].

### 2.2. Knowledge-Based Systems in Medicine

Medicine is one of the fields to have benefited most from the use of computers, as a pioneer in the use of Knowledge Based Systems [18]. Two landmarks that showed the validity of these technologies in medicine are ELIZA and MYCIN. The laboratories of the Massachusetts Institute of Technology (MIT) developed in the 1960s the ELIZA project, simulating the behavior of a psychoanalyst. This was the first application in psychiatry that offered a “credible” answer to patients' questions by constructing generated sentences to these questions by changing certain words or phrases. In the mid-1970s, the MYCIN project emerged, and over time it became one of the most influential systems in the history of KBS [19,20]. MYCIN was a system designed for diagnostics and therapy of infectious blood diseases.

The success of aforementioned past experiences positively contributed to the use of these technologies in medicine and healthcare. Table 1 contains a list of applications to support decision-making on monitoring of multiple diseases, including the name of the system, a brief description of its use and the date and country of creation [21]. As we may observe, only the LISA project and the SimulConsult tool are addressed to detect health problems in children. SimulConsult can perform neurological evaluations of children. In our study of KBS in medicine, no tool was found for the early detection of language disorders in children.

Carretero Díaz [22] defined an Expert System (ES) as “a system that solves problems in a certain area, with the skill and precision of an expert” in [20,23] they can find other definitions of these type of systems. An ES is an ideal system in order that the experts share knowledge with not expert people, therefore the system must get up-to-date constant on the part of experts which will allow that it should be used by specialists who do not have sufficient knowledge in the area of application. The most important advantages of an ES in order to contribute with regard to other information systems are [24]:

- It can support the decisions of many persons simultaneously thanks to the system's terminals (a human expert cannot to be available at all time and in several places at the same time).
- It can improve the productivity of the system (minor time of response).
- It can provide stability and consistency to the process of decision in a certain area.
- It reduces the dependence opposite to the employees (for vacations, to leave working places, *etc.*).
- End with the shortage of available experts and reduce the cost of access to the knowledge of the experts.
- It is an excellent tool of training, it justifies the decisions.

## 3. Methods, Materials and Design

### 3.1. Methods

The acquisition and systematization of needed knowledge required for the early referral KBS of a smart system proposed in this paper is a critical aspect that determines its effective use in primary care. The process of Knowledge Acquisition (KA) is the first step for creating a KBS and it strongly influences the conditions for correct operation. This process covers up to the final stage of KBS development.

#### 3.1.1. Method to Knowledge Acquisition and Formalization

The methodology for KA requires consideration of both the definition of the knowledge to be systematized and the conceptualization and formal design of the information compiled from human and materials sources in order to model the functioning of the KBS. For this reason, the main methodologies available for extracting knowledge were studied, with a comparison of GROVER, CommonKADS (CK), Methontology and IDEAL. The conclusion of this study was to use a combination of CK and Methontology, as these methods offer the greatest potential for application in certain phases of the KBS construction. The relation between the KA collected from humans' expertise and its translation into a formal ontology is successfully achieved in a cyclic way.

CommonKADS is a knowledge engineering methodology for the design and development of KBS based on knowledge extracted from human experts and its codification to allow for its processing by a system [20]. The application of CK for the system design provided a set of early detection items to be considered by the pediatrician. This structured knowledge reflects all important aspects of the KBS to be implemented and verified through a user tool. CK was used in KA meetings as it is most suitable for modeling the knowledge extracted from language specialists in the form of ontology.

Methontology is a methodology oriented towards the implementation of ontology in the activity of conceptualization of the KBS that has been successfully used by many authors [25]. Methontology defines a set of tasks that enabled moving from an informal specification of the domain of application, collected with the language specialists, to a semi-formal specification of the domain. This facility makes easier the understanding of the ontology for smart screening of language disorders by consulted neuropediatricians and language therapists as well as the system developer.

One key point to guarantee the success of the KBS construction is the selection of a suitable team of experts. Next, the members of the team of experts are presented:

- A neonatologist with high expertise in development disorders and child disability, former director of the neonatology department of San Carlos Hospital in Madrid,
- Five primary care pediatricians, in two Primary Attention Centers,
- A neuro-pediatrician presently working in the Quirón Hospital of Madrid,
- Two experts in specific language impairment who are therapists at the Language Intervention Center (LIC) at La Salle Campus (UAM) of Madrid.
- A therapist working at Legamar School.

#### 3.1.2. Knowledge Base System Development

**Figure 1.**
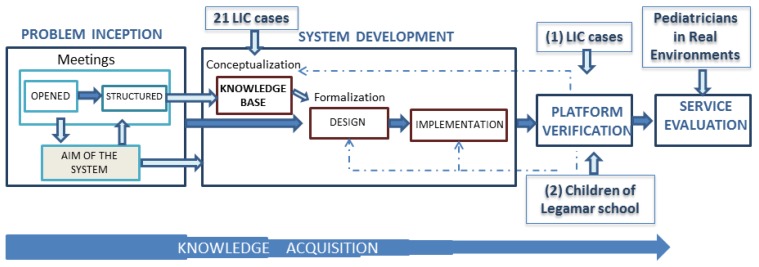
Knowledge Base System Development Methodology. This figure summarizes the empirical design methodology for the construction of the KBS (Pegasus).

Once the objectives, methods, and experts have been identified a strategy for KBS development is needed. Figure 1 shows the whole process for the development and validation of the final solution.

KBS development has been carried out in five steps:

- **Problem Inception**: its aim is to define the problem to be solved for early intervention. The group of experts involved in the KA process in this phase were a pediatrician, a neuropediatrician and a neonatologist. Five open meetings, held between September 2009 and May 2011, supported the problem definition and led to specifically work with early screening of language disorders, by the construction of a smart detection system.
- **System Development**: it covers the implementation phase of the KBS and its core KB. In this phase, the group of experts included a neuropediatrician and two therapists in specific language impairment who work at the Language Intervention Center (LIC) at La Salle Campus (UAM) of Madrid. They are experts on neurological disorders in children and helped to create the KB through ten structured meetings. The process of building and purging the KB was developed through a retrospective analysis of information on levels of language acquisition of 21 children who received therapy in the LIC [26]. The KB has been developed using Protégé (ontology creation platform) [27] and Pellet (reasoning engine) [28]. The verification of the KB required a usable tool so that specialists might interact with the KBS in an efficient way.
- **Platform Verification**: experts should be able to evaluate whether the system proposal to refer to a specialist arising from a detection of language development disorders was correct or not. This process contributed in a satisfactory way to improve the graphical user interface and the reasoning rules of the KB. A web tool (Gades) has been built in the verification stage to facilitate the work of experts in specific language impairment who are working at the LIC and a therapist working at Legamar School [29]. This phase has two planned stages:
  1. KB verification by means of children's retrospective cases who have received therapy in the LIC. This process of check it has fulfilled Mrs. Maria Peñafiel, who until August, 2012 was working as therapist of the LIC and currently she is a principal in the Legamar School. The 21 cases used in this stage differ with regard to the conceptualization stage. The obtained results are gathered in the results chapter. These results will be used for the iterative refinement of the KB in next steps.
  2. KB verification by means of children's Legamar School. This checking stages runs from March to September, 2013 along six months. This validation aims to get ready for the summative evaluation to be performed in clinical routine with all kinds of children having a normative development or with a linguistic or development delay.
- **Service Evaluation**: this controlled evaluation is scheduled for autumn 2013 in cases considered of interest by the five pediatricians already involved in the final stage. Thus, a web tool (Pegasus) is being built to facilitate the work of experts and primary care pediatricians. Six doctors (five pediatricians and one neuropediatrician) will be involve in the verification stages both for usability and system performance tests along six months. End users pointed out that they could use the tool by themselves in clinical routine.
- **Knowledge Acquisition**: acquisition and formalization processes were developed on the basis of information gathered in open meetings and then structured with the team of experts. The process of acquisition of expert knowledge relied on the use of additional techniques such as questionnaires, surveys and interviews designed in accordance with the objectives to be met by the smart system, with a view to support pediatricians in primary care working in the public health system.

Knowledge Representation (KR) is the first task to take into account to develop a system. KR must globally consider the transformations of the web interface, *i.e.*, changes in the web site structure and content, as well as user interaction models. It is not a trivial task to find out an effective KR method. Velasquez and Palade suggested a Knowledge Base (KB) creation methodology to implement a web-based computerized recommendation system [30]. The information of the KB is a key part in the KBS and this work presents the KB result in the section four.

### 3.2. Materials

Children's data involved in the KA process and KB verification came from 21 LIC cases. Table 2 outlines ratio of children who could not speak or had unclear speech that were seen by the pediatrician as requested by their parents. Professionals sounded alarms by themselves along routine child visits.

The development of the Gades web tool supported the verification process with retrospective cases from LIC. Experts benefit from this tool to validate the responses of the system for the developmental items provided by the KB [29]. Primary care pediatricians will validate the KB on autumn 2013 by using an *ad-hoc* web interface called the Pegasus web tool.

The knowledge extracted in meetings should be represented in a well-structured and comprehensible manner in a KB to make the knowledge useful and relatable for solving problems arising in the domain of execution. The solution of problems detected in the execution domain request a KR of data acquired in meetings. A well-structured and comprehensible manner is needed to provide useful and relatable knowledge through the KB. As noted by Torsun in 1995, KR requires formalisms or structures that represent it, either through declarative logic, mathematical formulas or concept maps [31]. The use of logical basis, as a tool for KR, requires adapting formal languages to models of knowledge expressed in natural language. Out of all the KR available models, this research used description logic as it directly supports the development of ontologies for integration in the semantic web, as it is done extensively with Ontology Web Language (OWL) language [32].

### 3.3. Functional Specification of the System (Design)

**Figure 2.**
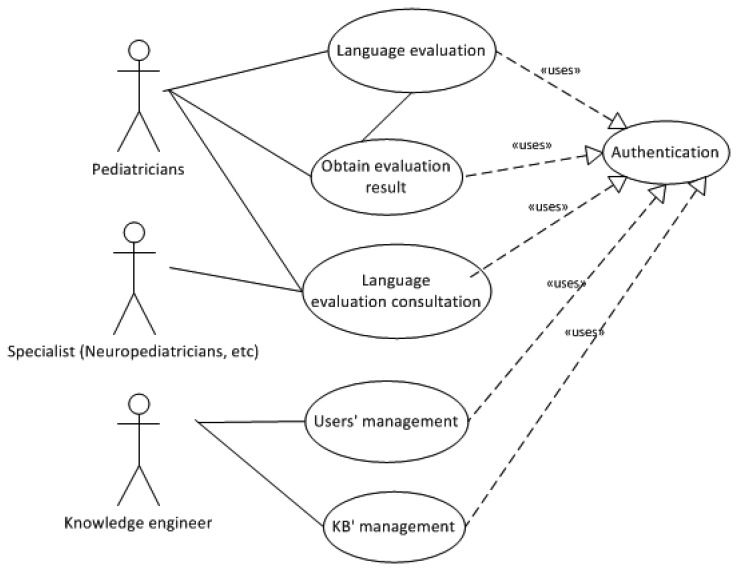
Pegasus general use case model.

Pegasus system was designed according to the use case model of Figure 2, written in Unified Modeling Language (UML). The KBS was modeled with this use cases diagram, which describes its proposed functionality as offered by the system. Main systems actors are:

- The primary attention pediatrician
- The specialist the child has been a derivative after the introduction of his case in the system for primary attention pediatrician
- The knowledge engineer entrusted to manage the information of the users of the system and of managing the information of the KB

A description of the actions for every use case is provided below (see Figure 2):

1. **Language Evaluation**: the primary care pediatrician will perform the language acquisition evaluation in a child. The pediatrician will connect to corresponding Web page and will update the information of the child requested by the system. The system will verify if the validity of this information and will ask for modifications in case of error.
2. **Obtain Evaluation Result**: when the introduced information is correct, the system shows question suggested to check the language status of the child corresponding with the child's age in months. If the state is normal the system will indicate it with a message, in other case the system can propose bringing forward visit (for re-evaluation of the level of language acquisition) or suggest referral to an appropriate specialist.
3. **Language Evaluation Consultation**: the specialist to whom the child is derived by his/her primary care pediatrician will be able to consult the study case information and verify the answers given by the pediatrician according to the age of the child. In this way, the specialist has information to support the diagnosis procurement tasks.
4. **Users' Management**: the knowledge engineer will be administrator of the information of the users with the different profiles that they can access to the system.
5. **KB' Management**: there will exist the possibility of modifying the information of the domain, in order to introduce new inferences that modify the reasoning of the KBS.

## 4. Development

This section contains the development of the research. First, it describes the general System Architecture. Secondly, it describes the construction of the KB and then the process of formalization of the KB in OWL with the tool Protégé.

### 4.1. General System Architecture (Pegasus Web Tool)

**Figure 3.**
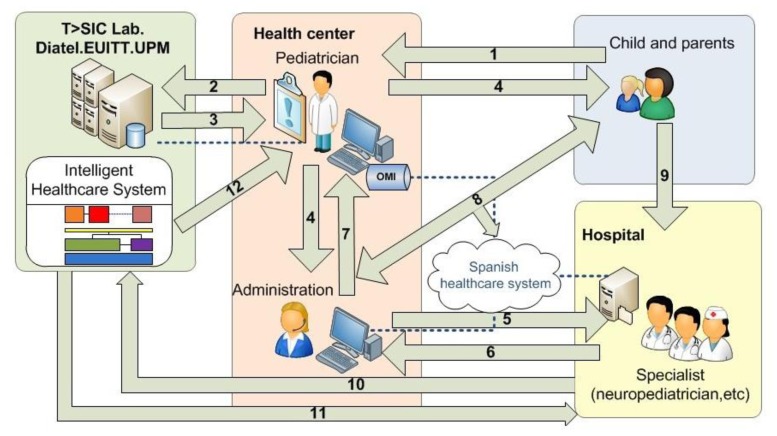
Pegasus general system architecture.

The functional architecture of the resulting system must facilitate dynamic interaction between the actors involved; distributed platforms for the management of information, models of reasoning and processes in line with the health care model in which it is located (see Figure 3).

Next a description of the steps given in Figure 3 is provided:

1. The child goes to the family pediatrician accompanied by a family member.
2. Primary health-care doctor decides to use the KBS to assess whether there is a language disorder in the child. The doctor will introduce the required information.
3. The KBS returns the result to the pediatrician. Three possibilities:
  - The result is that everything is normal.
  - The result changes the visitation schedule the child's pediatrician.
  - The KBS proposed to be derived from the relevant specialist hospital. In this case the doctor decides whether to accept the decision of KBS and pursue the request for appointment at the hospital, or decide it is not necessary.
    As always the KBS decision supports the pediatrician, but is never an imposition on the decisions that the doctor makes.
4. Health Center Administration requests an appointment with the specialist.
5. An appointment with the specialist is requested from the hospital.
6. Response is received to the request for appointment at the hospital,
7. The details of the appointment with the specialist will be received by the pediatrician.
8. The appointment details reach the child and his family.
9. The child goes to the specialist.
10. The specialist checks the KBS response for the corresponding case.
11. The DSS returns the result of the evaluation process for that case.
12. The DSS notifies the pediatrician that the specialist has accessed to the system. The specialist will validate the KBS outcome in order to improve the KB.

### 4.2. Knowledge Base (KB) for Language Disorders

The KB has been built through an iterative process of structured meetings between September 2011 and May 2012. Only the two language therapists and the neuro-pediatrician participated in the meetings, using CK techniques to extract information such as structured interviews to complete the KB with questions to be asked by the primary care pediatrician children's tutor upon arriving at the care facility. The starting point for building the KB is the Denver Test, as it is extensively used in primary care [33].

With this basis, questions were sharpened to focus on language, with a view to enabling the primary care pediatrician to detect possible delays in children's development that require closer attention or immediate referral to an appropriate specialist. The process of building and purging the KB is based on the experience of LIC, who checked developmental items against the appearance of language disorders through a retrospective analysis of information on levels of language acquisition of 21 children who received therapy in the LIC [26]. In materials sub-section summarizes the data of the 21 LIC cases analyzed in the KA process (Table 2), which lays the groundwork for the KB by indicating the most frequent alarm periods in detection of disorders and the resulting diagnosis.

Observation of the data gets back to the hypothesis that diagnosis in all cases is related to the level of language acquisition, or problems deriving from it, when children begin to read and write. In most cases, it is the family which belatedly detects delays in language when a child barely speaks at the age of two years. In two of the 21 cases, parents did not become alarmed until the child has surpassed the age of three years. Information systematized with the LIC therapists is significant for mastering the problem [26] and enabled relating KB questions with the demand for intervention in cases of either alert or alarm.

The study showed that the questions would have a negative answer in many of the cases under study and, for this reason; therapists consider them to be significant. The structuring of the final KB consists of 136 questions between month 1 and month 72 in the life of the child, and questions may be of two main types:

- Questions called Alert Milestones that imply bringing forward the visit. A negative answer to these developmental milestones means that the child makes return visit within three months to allow for re-evaluation of the level of language acquisition.
- Questions called Alarm Milestones that imply referral. These items could be considered as reasons for alarm and suggest referral to an appropriate specialist.

Table 3 details the KB questions for a child between the ages of 1 and 4 months. The first column indicates the child's age in months at the time of evaluation and the question type (Alert or Alarm). The second column shows the question the pediatrician asks the child's tutor to evaluate the child's state of language acquisition and the “System decision” column contains the system's answer in the event of a negative answer to the question (referral to specialist or bring forward visit).

### 4.3. Formalization of Primary Care KB in OWL

As noted by Hervas *et al.*, “An Ontology can be specified through several formal mechanisms”. In order to allow the computation of the conceptual knowledge gathered from experts a formal representation is needed [34]. One common approach to represent ontological concepts is using basic modeling languages such as entity-relationship model. More powerful languages, as frame-based languages, allow the definition of concepts and relationships. Currently, the most expressive languages are based on logic-based models, as First-order Logic for example, which allow the specification of concepts, relationships and restrictions. The challenge to choose one or another language is the agreement between expressiveness and computability. Next, the formalization process of the conceptualization model is presented.

In the revision carried out for this research, the construction of the ontology according to Methontology required categorizing the questions the pediatrician must ask according to the months of age of the child at the time of evaluation.

Protégé was used in formalization of the knowledge model to create the KB and the inference engine needed to support decision making. Protégé offers an open and useful environment for the design, modeling, implementation, manipulation and viewing of ontologies.

**Figure 4.**
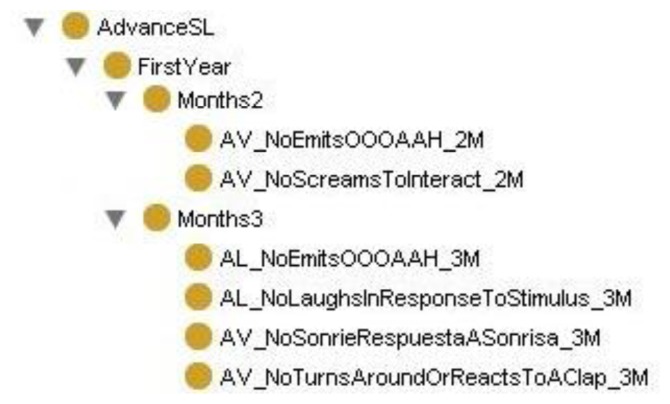
Ontology of the system in Protégé. This figure shows Ontology of system in Protégé for the classes of months 2 and 3.

The ontology of Protégé was built with a class hierarchy for the first six years, including a sub-hierarchy of classes for each month corresponding to the questions to be asked by the pediatrician. The class hierarchy in AvanceSL, in each month, includes the questions for each month as classes, as shown in Figure 4 for months 2 and 3 of the first year. The OWL of the binary relationships between ontology classes is defined as follows:
<owl:class rdf:ID=“**NextVisitInThreeMonths**”> <rdfs:subClassOf rdf:resource=“#Year_1”/>  <owl:equivalentClass>   <owl:Restriction>    <owl:someValuesFrom>     <owl:Class>      <owl:unionOf rdf:parseType=“Collection”>       <owl:Class rdf:ID=“**AV_NoEmitsOOOAAH_2M**”/>       <owl:Class rdf:ID=“**AV_NoScreamsToInteract_2M**”/>      </owl:unionOf>     </owl:Class>    </owl:someValuesFrom>    <owl:onProperty>      <owl:ObjectProperty rdf:about=“#**ThereIsNegativeResponseIn”**/>    </owl:onProperty>   </owl:Restriction>  </owl:equivalentClass></owl:Class>

**Figure 5.**
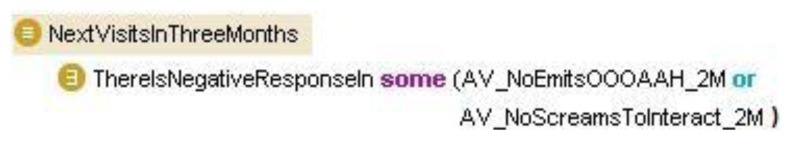
Inference in Protégé for questions about 2 month-old child.

The definition in OWL of the binary relationships established between ontology classes will sustain the system's reasoning process through axioms such as: If the child is 2 months old and we get a negative answer to the question: “Emits OOO/AAH” or “Screams to interact”, then “To anticipate the next visit in three months” (see Figure 5).

**Figure 6.**
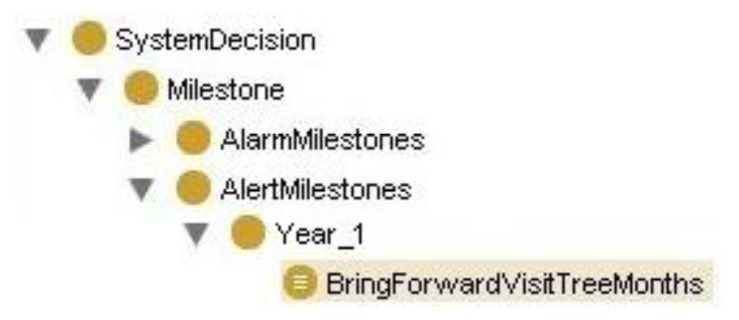
Inference in Protégé for questions about 2 month-old child.

Hence, the class hierarchy has been created to make inferences through the Decision System class, where this class contains motor decisions according to year and the type of milestone to which the decision belongs. Figure 6 shows the logical formulation of the correspondence of these axioms with inferences in Protégé.

## 5. Deployment

On the one hand, the final application to be developed is a Web type, so the commands the interface generates will have to be as small as possible, on the other hand the system (hardware and software) the pediatrician uses must be transparent and fast. Thus, the architecture of the platform must be modular and distributed to allow the reutilization of the modules that compose the solution as well as the interaction with the developed ontology. Java EE is a component architecture whose utilisation is justified by its open communication modules to support and scale such an approach. It provides a set of classes and interfaces to communicate with the requested database and supports routing of requests that help handle mistakes, control its reliability and manage events. Java EE also has a mature development of these architectures and it is widely accepted in the health care domain, compared with emerging technologies like HTML 5. In what refers to the database, there is a need of a multithread and multiuser server that is robust and fast. The motives are exposed in the previous point, it is necessary to provide service to the users who connect and work with sensitive information. MySQL expires with these requirements and fosters the creation of relational databases that can be consulted across the standard commands of MySQL. The technology is developed based on a generic client-server model, where the client is modeled, placed outside of the network, and realizes requests to the server. These requests are realized across URLS's introduction in a browser installed in the client and are sent to the server by means of the HTTP protocol. The following deployment graph shows the physical relations between the hardware and software components of the system.

### 5.1. Gades Web Tool Deployment

**Figure 7.**
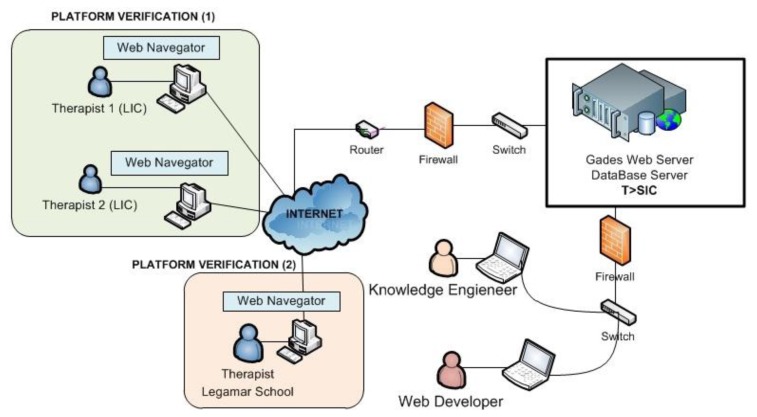
Gades web tool deployment.

The system platform technology of Gades has been developed at the Group “Telematics Systems for the Information and Knowledge Society”: T > SIC (Universidad Politécnica de Madrid), the developed system has been established in one of the servers of the group. The Knowledge Engineer tested the Gades functioning to verify the system with the proof cases of thirty children for three weeks. The different users access the Gades system to realize the process of requested evaluation across Internet (see Figure 7).

This phase was planned through two incremental phases:

- Platform Verification (1): KB verification by means of children's retrospective cases that have received therapy in the LIC.
- Platform Verification (2): KB verification by means of children's Legamar School.

**Figure 8.**
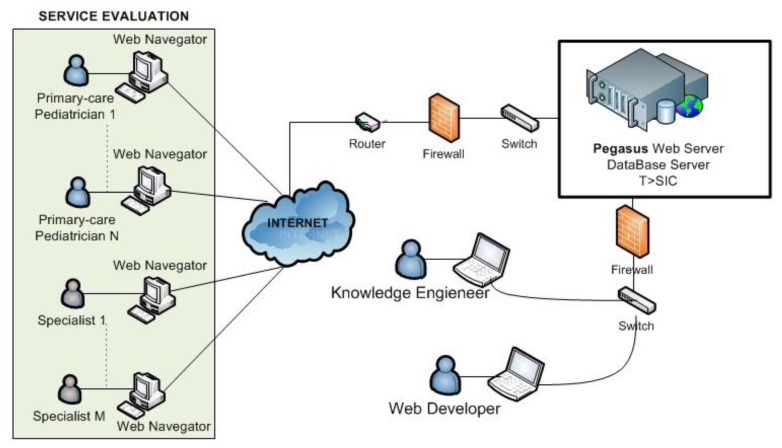
Pegasus web tool deployment.

The system platform technology of Pegasus will be deployed in one of the servers of the Group “Telematics Systems for the Information and Knowledge Society”: T > SIC (Universidad Politécnica de Madrid) as shown Figure 8.

The service evaluation is scheduled for autumn 2013 in cases considered of interest by the five pediatricians already involved in the final stage.

## 6. Platform Verification

**Figure 9.**
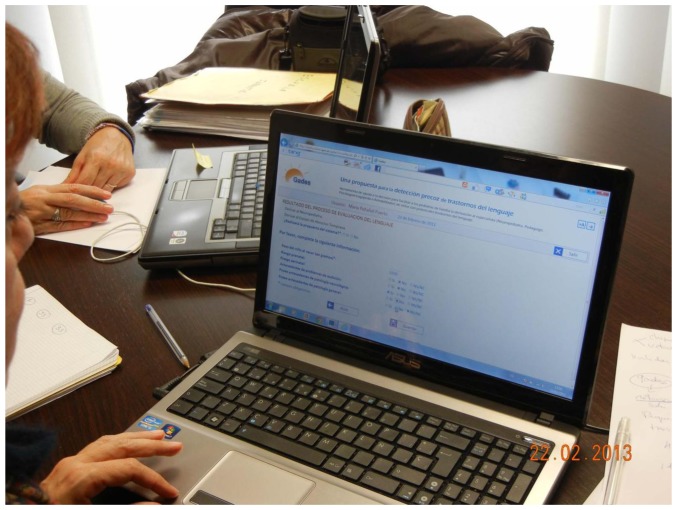
Language therapist realizing the system verification (Gades).

An example of an *ad-hoc* web interface (Gades) is shown for verification of the resulting KBS in this section. In a first stage, the solution has been tested by a therapist expert in specific language impairment with children's retrospective cases who have received therapy in the LIC (see Figure 9).

### 6.1. KB Verification through a Primary Care Tool (Gades Web Tool)

**Figure 10.**
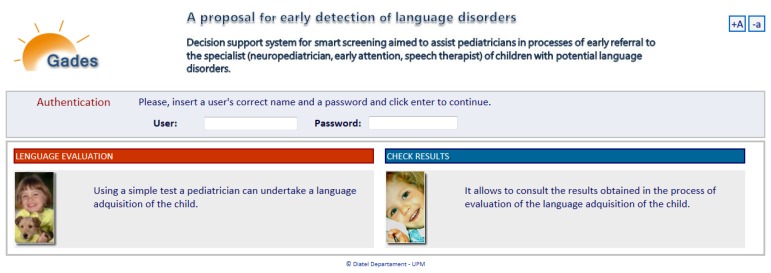
Access to the early detection tool of language disorders.

The verification of the KB required a usable tool so that specialists might interact with the KBS in an efficient way. Experts should be able to evaluate whether the system proposal to refer to a specialist arising from a detection of language development disorders was correct or not. Thus, a web tool was built to facilitate the work of experts and primary care pediatricians. For this reason, the Pegasus web system is being deployed. Meanwhile the Gades web tool provides the final users (two therapists) with a usable verification interface as shown in Figure 10 [29]. The resulting tool is based on an internal connection of the KB, implemented in OWL, with a Pellet reasoning engine.

This phase has been planned in two stages:

1. KB verification by means of children's retrospective cases that have received therapy in the LIC. The obtained results are gathered in this section. These results will be used for the refinement of the KB.
2. KB verification by means of children's Legamar School. This process of checking initiates in March, 2013, and it has a foreseen duration of six months.

**Figure 11.**
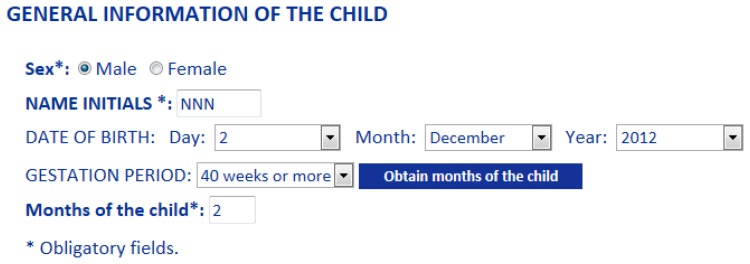
Gades process of language evaluation. General information of the child for starting the process of language evaluation.

Three language therapists were involved in the verification stages both for usability and system performance tests along six months. This process satisfactorily contributed to improve the graphical user interface (Gades) and the reasoning rules of the KB. Pediatricians pointed out that they could use the Pegasus tool by themselves in clinical routine. Once the language therapists logged into the Gades system (Figure 10), they began to evaluate a child's state of language acquisition starting from the general information (Sex, Name Initials, Date of birth, Gestation Period), as shown in Figure 11, and updating sensitive variables identified in literature such as weight, presence of hearing loss, previous neurological and general pathologies. This process was performed in a random and anonymous way with samples which are homologous to the original cases that were considered from the LIC records.

**Figure 12.**
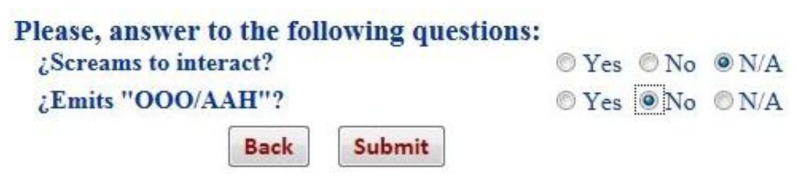
Process and result of language evaluation.

**Figure 13.**
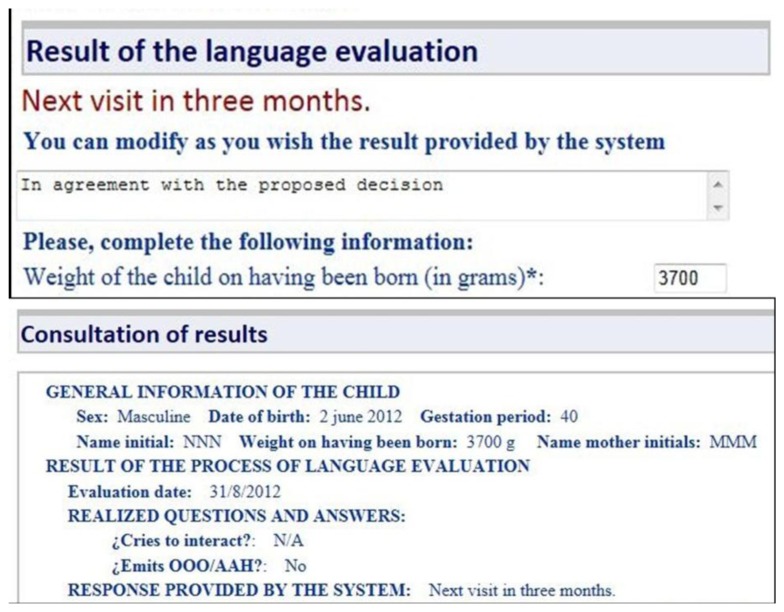
Query of results of language evaluation.

Figure 12 shows a sample of the question suggested by the system to check the language status of a two months baby: “Emits OOO/AAH” and “Screams to interact”. Once the pediatrician answers these questions, the result is provided by the early detection tool based on a negative answer to the question “Emits OOO/AAH”. At this point, the system proposes bringing forward the next visit and also suggests that the pediatrician enter an opinion on the system's decision or an alternative to the proposal of the system as detailed in Figure 13.

After the language evaluation process, the pediatrician or specialist—speech-language pathologist, neuropediatrician, early attention—to whom the child is referred did access to system to check the results of the language evaluation. Figure 13 shows the consultation of results as provided by the KBS in this step. This facility made possible the positive verification of the smart system for language disorders screening according the criteria of the five medical users involved.

### 6.2. Results and Discussion

The Gades system has been verified by a therapist from LIC with retrospective cases of 21 children who were treated at the center.

A review of the KB is suggested to get a more accurate response in order to derivate to different professionals (speech therapist, language therapist, neuropediatrician). The evaluation of the Gades solution has been performed by retrospectives cases of children who receive therapy at LIC. The cases consider two comparable different segments:

(a) Cases with normal development (14% corresponding to subjects 11, 12 and 14 in Table 4). These children were treated at LIC but the evaluation process of language skill was positive so they were not diagnosed with language pathology. The involvement of healthy children allows validating the system to avoid false positives. Paediatricians in real environments will work with children with normal development in the validation stage what justifies this system verification to correctly discriminate presence or absence of possible disorders.
(b) Cases of children who were diagnosed with a linguistic delay (86% of the cases, the remaining subjects in Table 4).

**Figure 14.**
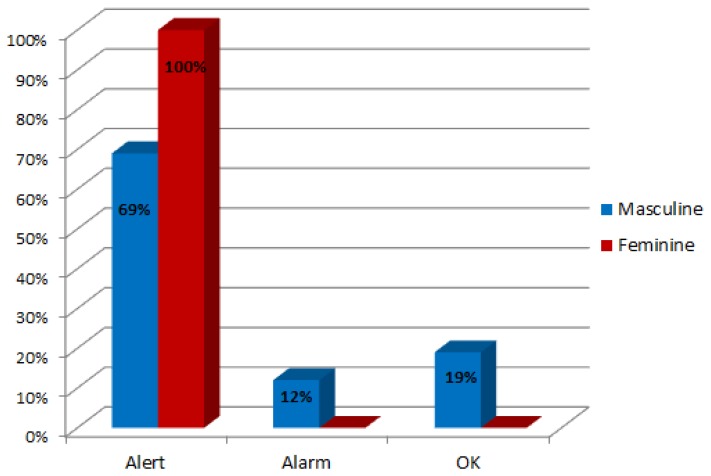
Results of language evaluation by gender.

Although gender is not the most important factor to state if the child is at risk of language disorder, the research showed a distribution of 24% female cases (subjects from 1 to 5) and 76% male. Since the subjects have been arbitrarily chosen, it could suggest that this pathology is more frequent in the male gender. This assertion should consider the data of Table 2 where the percentage of male gender was higher than the female (66% of male subjects). The results of the language evaluation by gender are compared in Figure 14.

**Figure 15.**
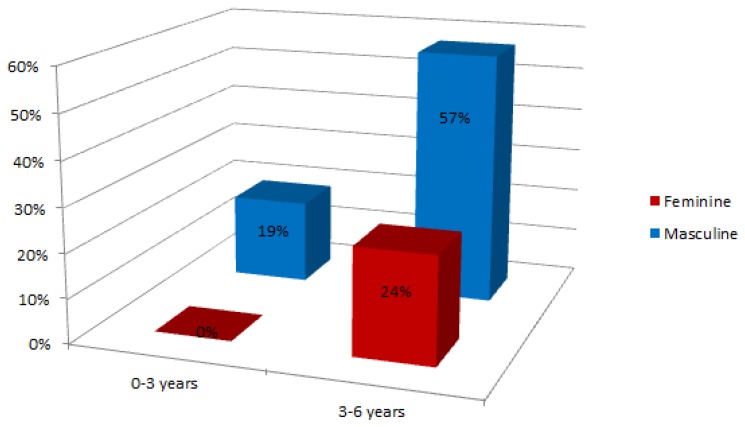
Language results in each stage of growth by gender.

As shown in Figure 14, Gades obtained an alert in the 100% of the female cases and in the 69% of the males. The system suggested an answer with an alarm event in the 12% of the male cases and with a positive evaluation in 19%. There are a low number of cases where the event obtained is an alarm. This situation is normal because all the subjects have been treated at LIC so the normal behaviour is to raise an alert. Figure 15 shows the percentage of cases that are in each stage of growth by gender.

There are few subjects in the range of 0–3 years (19% of the subjects). That is because the population under study is currently receiving treatment for language pathologies. This percentage reaffirms the need for a tool to early detection of language disorders.

Further to language difficulties and cognitive factors, this research revealed that the consultation for a first alarm in language acquisition delay happens at two years old in 35% of the cases and at two and a half years old in 40% [26]. 90% of the analysed children did not speak or just emitted single words and 10% did not understand or pay attention to external actions.

## 7. Conclusions and Future Works

This paper details an innovative smart solution to support efficient detection of language disorders among children aged 0 to 6 years in routine visits to pediatricians in primary care.

The research described in this paper shows the process of creation of a KBS to support the early detection of language disorders in primary care. Experimental results allow concluding that this solution effectively supports decision taking in pediatrics care in relation with early referral procedures. The efficiency of the system makes it ready both to detect cases that require specialised intervention and to use it at regular clinical routine. Both scenarios, that is to say, use for early detection sessions or regular visits, need to be complemented by detailed diagnosis procedures in the case of referral decisions taken by the pediatrician.

The combined use of CommonKADS and Methontology facilitated the construction and formalization of an ontology that enables the implementation of an intelligent system capable of tackling the problem both in an efficient and effective way. The involvement of four experts in neuropediatrics, neonatology and language disorders led to define the problem and select significant 21 real, proven cases, from a base of about 60 clinical records. Next, the KB was fine-tuned and verified thanks to available experience of five pediatric care specialists. The KB, formalized with the resulting ontology, has shown its potentiality to assist pediatricians in detecting language disorders. The research is validated through its verification stage with 21 cases obtained from a center oriented to the detection and treatment of language disorders (LIC). These cases were not used in the formalization stage in order to fine-tune the system with the Gades tool.

The results of this initial verification process shows success in 100% of the cases according to the response provided by the system for each case study. Children with positive evaluation, without language disorders, were included in the study to check the validity of the results. The response of the system is also consistent with the clinical expectations.

The development and evaluation process also reflects that there are a greater number of cases of boys than girls. This leads to the conclusion that such diseases happens very often on male gender.

The opinion of the experts involved allows for a forthcoming start of the second stage of validation, with a view to evaluation in longer routine clinical practice. This controlled evaluation is scheduled for autumn 2013 with cases considered of interest by the five pediatricians already involved in the final stage. A future line of research would be the design of a model of self-learning based on unsuccessful referral information that can be compiled in the fine tuning planned for the system in the deployment phase in primary care. The design and implementation of the KB realized for early detection of language disorders will be used to build up other detection systems of diverse neurological disorders in children, with the revision of the KB and the participation of some experts in this disorders.

Since the KBS has been satisfactorily validated by health care professionals, future research aims to design an usable mechanism in order to optimize the questions set and their associated factors according to the answers gathered from the children's cases and the KB. Authorized actors involved in the process might create, modify and delete questions, associated factors and relationships, according to a scientific agreement model. In this way, the optimization of involved questions and factors could help to increase the accuracy of detection. Currently, it is planned to evaluate the system in an additional health care center and in a school for children with special educational needs. Other development disorders related to language are being taken into account, such us potential delays in motor and sensor abilities.

## Figures and Tables

**Table 1.** Support systems for decision-making in medicine.

**System Name**	**Description**	**Date and Place**
HEPAXPERT I,II,III	Analyzes and interprets tests for detecting hepatitis A, B, C and D.	1991 Austria
VIE-PNN	SE for nutrition of newborn children in intensive care.	1993 Austria
CEMS	Supporting system for decision-making in mental health. Can be used for diagnostics and treatment of patients and monitors and gives alerts on methods and results.	1993 US
Coulter^®^ FACULTYTM	KBS used to assist in work flow as an educational tool in hematology laboratories.	1996 UK
TxDENT	Follow-up and provision of recommendations for patients undergoing odontological care	1997 Canada
RetroGram	Generates medication regimes using medical history and genetic information of patients with HIV	1999 UK
Automedon	KBS for administration of mechanical respiration in intensive care	2001 France
TherapyEdge	Graphically tracks and automatically processes information (medication, condition) of patients with HIV and chronic illnesses	2001 US
ERA	Support system for interactive decision-making for identifying patients suspected of having cancer	2001 UK
ATENÍA	Control of hypertension in primary care, offering recommendations for care and medication	2002 US
LISA	Assists in decision-making for children with lymphoblastic leukemia	2004 UK
SimulConsult	Support software for medical decision making allowing professionals to combine clinical and laboratory conclusions, allowing for identification of useful conclusions for a diagnosis	2008 US

**Table 2.** Analysis of the 21 Language Intervention Center (LIC) cases used for refinement of the KB.

**Sex**	66% boys and 29% girls
**First Alarm Sign**	66% cause alarm aged between 24 and 36 months because they do not speak 14% cause alarm aged more than 36 months because they do not speak 10% cause alarm owing to febrile seizure aged less than one year
**Person Who Sounds the Alarm**	90.4%: parents 4.76% of cases, alarm sounded by school 4.76% of cases, alarm sounded by pediatrician
**Diagnosis**	66.6%: specific language impairment (SLI) 28.6% delayed reading/writing (related to spelling difficulties) 4.76% cognitive delay (understanding of symbolic and verbal concepts)

**Table 3.** Shows KB questions for 1, 2, 3 and 4 months.

**Age—Milestone**	**Question to Be Answered by Pediatrician**	**System Decision**
1 month—Alarm	Reacts to a bell	Send to specialist to check hearing
1 month—Alarm	Vocalizes without crying	Send to specialist to check hearing
2 months—Alert	Emits “OOO/AAH”	Bring forward visit (three months)
2 months—Alert	Screams to interact	Bring forward visit (three months)
3 months—Alert	Turns around or reacts (closing eyes) to a clap	Bring forward visit (three months)
3 months—Alert	Turns at the sound of mother's voice	Bring forward visit (three months)
3 months—Alarm	Emits “OOO/AAH”	Check if hearing problem ruled out Refer to neuropediatrician
3 months—Alarm	Laughs in response to stimulus	Refer to neuropediatrician
4 months—Alert	Turns at the sound of mother's voice	Bring forward visit (two months)
4 months—Alert	Emits guttural sounds (AJOS)	Bring forward visit (two months)

**Table 4.** Results summary. This table contains a summary of the results obtained in the evaluation process.

	**Sex** **(1)**	**Child Months / Questions Months** **(2)**	**Result** **(3)**	Result Proposed by Gades * (4)	**Therapist Accepts (5)**	
**1**	Feminine	39 / 36	**Alarm**	**E.A. – N.**	Yes
**2**	Feminine	52 / 48	**Alarm**	**E.A. – N.**	Yes **
**3**	Feminine	59 / 54	**Alarm**	**E.A. – N.**	Yes **
**4**	Feminine	60	**Alarm**	**E.A. – N. – L.T.**	Yes
**5**	Feminine	60	**Alarm**	**E.A. – N.**	Yes
**6**	Masculine	27 / 26	**Alarm**	**E.A. – N.**	Yes
**7**	Masculine	33	**Alert**	**Bring forward visit (1 month)**	Yes
**8**	Masculine	35 / 33	**Alert**	**Bring forward visit (1 month)**	Yes
**9**	Masculine	36	**Alarm**	**E.A. – NeuroP**	Yes
**10**	Masculine	39 / 36	**Alarm**	**E.A. – N.**	Yes
**11**	Masculine	48	**OK**	**Language Acquisition OK**	Yes
**12**	Masculine	48	**OK**	**Language Acquisition OK**	Yes
**13**	Masculine	48	**Alarm**	**E.A. – N.**	Yes **
**14**	Masculine	52 / 48	**OK**	**Language Acquisition OK**	Yes
**15**	Masculine	60	**Alarm**	**E.A. – N.**	Yes
**16**	Masculine	60	**Alarm**	**E.A. – N.**	Yes
**17**	Masculine	60	**Alarm**	**Language Therapist**	Yes
**18**	Masculine	60	**Alarm**	**E.A. – N.**	Yes
**19**	Masculine	60	**Alarm**	**E.A. – N. – L.T.**	Yes
**20**	Masculine	72	**Alarm**	**E.A. – N.**	Yes
**21**	Masculine	72	**Alarm**	**E.A. – N.**	Yes

* Result proposed by Gades. E.A.: Refer to Early Attention; N.: Refer to Neuropediatrician; L.T.: Refer to Language Therapist;

** The therapist proposes for these cases (subjects 2, 3 and 13) only the referral to a speech therapist.
